# Acute medications’ intake for migraine: a one-year report in patients undergoing first evaluation at a third level Italian headache center

**DOI:** 10.3389/fneur.2024.1450039

**Published:** 2024-08-29

**Authors:** Adriano Bonura, Alessandro Alesina, Elisabetta Sapio, Nicoletta Brunelli, Marilena Marcosano, Claudia Altamura, Fabrizio Vernieri

**Affiliations:** ^1^Neurology Unit, Università Campus Bio-Medico di Roma, Rome, Italy; ^2^Headache and Neurosonology Unit, Fondazione Policlinico Campus Bio-Medico, Rome, Italy

**Keywords:** migraine, NSAIDs (non-steroidal anti-inflammatory drugs), migraine prophylaxis, CGRP (receptor) monoclonal antibodies, medication overuse headache (MOH)

## Abstract

**Background:**

Headache disorders, particularly primary headaches like migraine and tension-type headache, still remain underdiagnosed and undertreated despite their high prevalence and significant impact on quality of life. In recent years, several specific medications targeting key pathways in the pathophysiology of migraine have been developed. Despite this advancement, numerous studies indicate that non-steroidal anti-inflammatory drugs (NSAIDs) and analgesics remain the most commonly used drugs. This study focused on the use of NSAIDs and simple analgesics as acute treatments for migraine among patients at a tertiary headache center.

**Methods:**

A retrospective observational study was conducted at the Fondazione Policlinico Universitario Campus Bio-Medico throughout 2022. Data were collected on the type and frequency of headaches, the usage and dosage of NSAIDs and other medications, and changes in their use at follow-up visits. Statistical analyses were performed to evaluate the efficacy and determinants of NSAID consumption and headache frequency changes.

**Results:**

Two hundred and eightythree patients diagnosed with migraine undergoing their first examination at our center were enrolled. Initially, 58.7% of patients used NSAIDs or simple analgesics, which decreased to 46.6% 3 months after, while triptan use increased from 65.1 to 72.8%. Changes in prophylactic therapies were significantly associated with a decrease in NSAID intake (*W* = 834.000, *p* = 0.004) and in headache frequency (*W* = 5960.5, *p* = 0.003). Specifically, the addition of topiramate or amitriptyline was associated with a reduction in NSAID use and headache frequency. Even pain freedom after the intake of NSAIDs improved from 55.2 to 79.4% of cases at follow-up.

**Conclusion:**

The study highlights the importance of appropriate diagnosis and tailored treatment strategies in the management of primary headaches. It underscores the need for specialized care to enhance treatment efficacy and patient outcomes, demonstrating that adjustments in prophylactic therapy can significantly reduce NSAID intake and improve headache care. This reinforces the role of tertiary headache centers in providing specialized care that can adapt treatments to individual patient needs and improve overall headache management.

## Introduction

Headache, a prevalent disease experienced by nearly 90% of the population at least once in their lifetime, poses a substantial health concern (1). The International Headache Society (IHS) has meticulously classified about 300 distinct types of headaches, categorizing them as either primary or secondary (2). Within the realm of primary headaches, migraine and tension-type headache stand out as the most prevalent, each presenting unique clinical characteristics and therapeutic challenges (2, 3).

Migraine, characterized by pulsating and often unilateral pain and accompanied by symptoms such as nausea, vomiting, photophobia, and phonophobia, can persist for durations ranging from 4 to 72 h (2). The pathophysiological basis of this condition points to the trigeminal sensory calcitonin gene-related peptide (CGRP) and its pivotal role in activating the trigemino-vascular pain pathway. The discovery of CGRP has led to profound changes in migraine therapy, ushering in the era of anti-CGRP pathway drugs, i.e., monoclonal antibodies and gepants (4). In contrast, tension-type headache, once predominantly considered psychogenic (5), now seems to reveal a neurobiological foundation (2). Despite being common and well-studied conditions, nowadays a minority of people with headache are diagnosed appropriately and on time by healthcare providers, and headache disorders are still underestimated and under treated throughout the world (6).

Pharmacological interventions for these two primary headaches typically encompass both acute and preventive treatments (3, 4, 7). Today, we have a wide spectrum of acute drug targets and classes with different mechanisms of action, efficacy, and safety profiles, including simple analgesics and non-steroidal anti-inflammatory drugs (NSAIDs), selective serotonin 5-HT1B/1D receptor agonists (triptans), CGRP receptor antagonists (gepants), and 5-HT1F receptor agonists (ditans).

Specifically, simple, non-opioid analgesics and NSAIDs emerge as fundamental options for the acute treatment of both migraine and tension-type headache, and they are still indicated as the first-line medication to treat migraine attacks (8–10). Despite the availability of more specific agents like triptans and gepants, NSAIDs still constitute a significant 52.6% of drugs employed for acute management, surpassing triptans at 33.6% (11). Although NSAIDs are not specifically designed for migraine treatment, they have demonstrated efficacy in alleviating mild to moderate pain intensity (8). Furthermore, their over-the-counter availability may explain their high prevalence of use (11). By inhibiting cyclooxygenase and reducing prostaglandin synthesis implicated in migraine pathophysiology, NSAIDs provide a widely accessible treatment option (12). However, the use of NSAIDs is not without drawbacks, as they are associated with adverse effects such as an increased risk of cardiovascular events, duodenal ulcers, and the potential for medication-overuse headache (13). Among simple analgesics, paracetamol primarily exerts its analgesic activity through an active metabolite, AM404 (N-arachidonoylaminophenol), which acts by inhibiting the reuptake of endocannabinoids in the synaptic space (14). Additionally, it exhibits a mild inhibitory effect on cyclooxygenases (15) (see Table 1). On the other end, triptans are the first-line therapy for the acute treatment of moderate to severe migraine (see Table 2). However, they are still underused, as at least half of patients suffering from primary headaches, including migraine, do not refer to primary or secondary headache services; thus, they do not receive a prescription for a specific drug (16).

**Table 1:** Pharmacocinetic profile and efficacy of analgesic and NSAIDs.

Drugs	Formulation	Tmax (h)	Half-life (h)	Usual dosage	NNT: 2-h pain relief	NNT: 2-h pain free	Repeated dose interval (h)	Max daily dose (mg)
Paracetamol	Tablets	0.5–1	2	1,000	5.0	12	4	4,000
Acetylsalicylic acid	Tablets	1–2	0.25 5–6 salicylic	1,000	4.9	8.1		5,400
	Effervescent granules	20 min		1,000			4–6	2,600
Ibuprofen	Tablets	1–2	2	400	3.2	7.2	4	2,400
	Solubile	<1	2	400				
Naproxen	Tablets	2	14	500–550	6.0	11	Twice a day	1,375
Diclofenac	Tablets	<1	2	50	6.2	8.9	3–4	150
	Powder for solution for oral use	15 min	2	50	5.1	7.4	Single	50

Data are extracted from Murmura et al. (26); Becker (27); Ong and De Felice (28).

**Table 2:** Pharmacocinetic profile and efficacy of triptans.

Triptan	Formulation	Dosage (mg)	tmax (h)	Onset (min)	*t*½ (h)	Biodisponibility %	NNT 2-h pain free	24-h relapse rate (%)
Sumatriptan	Subcutaneous	6	0.2	10–15	2	97	2.3	34–38
	Tablets	100–50	2–2.5	30–60	2	14	4.7–6.1	32
	Nasal spray	20	1–1.5	15–30	2	17	4.7	32–34
	Rectal	25		30–60				
Zolmitriptan	Tablets	2.5–5	1.5–2	45	2.5–3	40–48	5.9	22–37
	Orodispersible	2.5–5	1.5–3.3		2.5–3	40–48	4.6	32
	Nasal spray	5	2	10–15	2.82	42		26
Rizatriptan	Tablets	5–10	1.2	30–120	2–3	45	3.1	30–47
	Orodispersible	10	1.6–2.5		2	45		NA
Almotriptan	Tablets	12.5	1.4–3.8	60–180	3.2–3.7	70–80	4.3	18–29
Eletriptan	Tablets	20–40	1–2	<60	3.6–5.5	50	4.5	19–30
	Tablets	80	1–2		3.6–5.5	50		<33
Frovatriptan	Tablets	2.5	2.0–4.0	60–120	25	22–30	8.5	7–25

Data are extracted from Murmura et al. (26); Becker (27); Ong and De Felice (28).

Primary headaches are associated with high disability, impaired quality of life, and not indifferent financial costs. This study aims to gather comprehensive data on the utilization of simple analgesics and NSAIDs as acute treatment for migraine with and without aura, among patients attending the Headache Center at the Fondazione Policlinico Campus Bio-Medico of Rome. The objective is to understand the prevalence of simple analgesic and NSAID use, the specific NSAIDs predominantly employed, and their respective dosages in a real-world setting. Additionally, the study seeks to elucidate the determinants associated with the use and effectiveness of NSAIDs.

## Methods

### Study population

We conducted a retrospective observational study, collecting data on patients suffering from primary headaches and their usage of acute medications, undergoing the first examination at the Headache Center of the Fondazione Policlinico Universitario Campus Bio-Medico from January 1 to December 31, 2022. Our third-level headache center gathers from primary-care physicians and secondary-care services, mainly hard-to-treat subjects, i.e., when headache treatments are ineffective or inappropriate (17), patients with chronic migraine or daily headaches in general, many of them affected by medication overuse headache (MOH) (18). Inclusion criteria comprised (i) age ≥ 18 years and (ii) a diagnosis of primary headache, either migraine or tension-type (iii) three-month follow-up visits. Exclusion criteria encompassed (i) the presence of other primary headaches (such as trigeminal autonomic cephalgias, TACs, or other primary headaches), secondary headaches, or headaches of undetermined cause.

### Data collection

We collected the anamnestic, clinical, and pharmacological data of the patients’ first visit (t0) from January to December 2022 and the follow-up visits 3 months later (t1). The gathered information included age, type of headache (episodic or chronic migraine, presence of aura, tension-type headache), headache frequency (days per month), Migraine Disability Assessment (MIDAS) questionnaire score, type of simple analgesic and/or NSAIDs used, dosage, and frequency of drug intake (number of assumption per month). The efficacy of the response to acute medications was assessed using a 3-level scale that includes categories such as no effect (0), pain relief (1), and pain freedom (2). Additionally, other medications were recorded, including triptans, oral prophylactic medications, onabotulinumtoxin-A, and anti-CGRP treatments, indicating the type and their use at both t0 and t1 (Yes or No).

### Statistical analysis

Quantitative variables were presented as means, standard deviations, and maximum and minimum values, while percentages and fractions were used for categorical variables. Inferential statistical analysis was performed to correlate therapeutic changes made between t0 and t1 with variations in clinical outcomes, expressed in terms of changes in monthly headache frequency and NSAID usage frequency (calculated as the difference between frequencies at t0 and t1) and changes in efficacy (calculated as a dichotomous variable, improvement, or no improvement). To evaluate differences between continuous variable between t0 and t1 a paired test with Wilcoxon was implemented. Differences between categorical variable at t0 and t1 was implemented with McNemar Test. The distribution of quantitative variables was assessed using the Shapiro-Wilks test, while association between quantitative and categorical variables were analysed with the Student’s *t*-test (for normally distributed variables) and the Mann–Whitney test (if the distribution was non-normal). Association between categorical variables were assessed using the chi-square test.

### Ethics committee approval

The institutional ethics committee granted ethical approval for the study, with the reference number [PAR 26.23 OSS, clinical studies number 2023.17]. This endorsement affirmed the study’s adherence to ethical standards and the protection of participant rights, enabling the commencement of this single-center retrospective observational study.

## Results

### Population demographic data

The results are summarized in Table 3. The study enrolled 283 patients with migraine, undergoing the first examination at our center in the considered period; 86% were female, 6.7% (19 patients) had a concomitant diagnosis of tension-type headache. Chronic migraine was diagnosed in 26.9% of the study population, while the presence of aura was reported in 13.4% of patients. At baseline (t0), migraine patients had an average monthly headache frequency of 10.9 days (± 7.8, range: 0–31), which reduced to 6.8 days per month (± 5.3, range: 1–31) at the three-month follow-up (*p* < 0.001). The MIDAS score was reported in 83 patients at t0, with a mean score of 41.8 ± 42.1 and in 93 patients at t1 (mean score 24.3 ± 25.7) (*p* < 0.001). For all patients, the t0 visit represented their initial consultation at a tertiary-level headache center; for 73 out of them, the examination was the first-ever visit for headache, and they were naïve to specific migraine treatment and prophylaxis medication.

**Table 3:** Population demographics, clinical and therapeutic characteristics.

Variable		Counts	Total	Percentage%
Female		246	283	86.9
Mean age		44.9 ± 13.8	283	
Tension type headache (concurrent diagnosis)		19	283	6.7
Chronic migraine		76	283	26.9
Aura		38	283	13.4
		
t0				
Headache frequency (month)		10.9 ± 7.8	255	
MIDAS		41.8 ± 42.1	83	
Triptans usage		184	283	65.1
Prophylactic therapies usage		128	283	45.2
Monoclonal antibodies usage		56	283	19.8
NSAIDs usage		166	283	58.7
NSAIDs frequency		10.2 ± 9.5	166	
NSAIDs efficacy	Ineffective	18	116	15.5
	Pain relief	34	116	29.3
	Pain freedom	64	116	55.2
		
t1				
Headache frequency (month)		6.8 ± 5.3	255	
MIDAS		24.3 ± 25.7	93	
Triptans usage		206	283	72.8
Prophylactic therapies usage		177	283	62.5
Monoclonal antibodies usage		74	283	26.2
NSAIDs usage		132	283	46.6
NSAIDs frequency		6.1 ± 6.1	132	
NSAIDs efficacy	Ineffective	6	63	9.5
	Pain relief	7	63	11.1
	Pain freedom	50	63	79.4

### Drug assumption data

At t0, 166 patients out of 283 (58.7%) used to take paracetamol and/or NSAIDs, averaging 10.2 administrations per month (± 7.8, range: 0–60); 65.1% of patients used to take triptans (averaging 9.0 ± 8.5 administrations per month), and 35.3% of patients took both. Thirty-nine (13.7% of all population) patients had a diagnosis of medication overuse (MO, equal or more than 15 NSAIDs per month or equal or more than 10 days of triptan usage per month). At t1, 132 patients (46.6%) used NSAIDs with a reduction of frequency to an average of 6.1 administrations per month (± 6.1, range: 0–37, *p* < 0.001). At t1, triptans were prescribed in 72.8% of patients with a reduction of usage per month (mean: 5.7 ± 5.8, *p* = 0.018). At t1, only 11 patients (3.8% of all population, *p* < 0.001) still had MO. The most commonly taken NSAID was ibuprofen (22.6%), with dosages of 600 mg (14.1%), 400 mg (13.2%), and 200 mg (2.7%); some patients used different dosages. Ketoprofen lysine salt was used by 22.6% of patients, mainly in the 80 mg formulation (19.9%). Paracetamol was utilized by 14.1% of subjects (1,000 mg 11.7%, 500 mg 3.7%). There was no evidence of serious collateral effects related to NSAIDs or simple analgesic usage.

Triptans specific utilization rates at t0 were: eletriptan (23.3%), almotriptan (13.1%), rizatriptan (12.7%), frovatriptan (7.8%), sumatriptan (7.1%), and zolmitriptan (1.1%). In 13 patients, the triptan was switched to another type at t1.

Prophylactic therapies were employed in 45.2% at t0 and increased to 62.5% at t1. At t0 0.7%(2 patients) of patients were under more than one prophylactic therapy, at t1 1.4% (4 patients) of patients. Amitriptyline (t0 = 13.1%, t1 = 17.3%, *p* = 0.058), topiramate (t0 = 5.7%, t1 = 12.7%, *p* < 0.001), and propranolol (t0 = 8.8%, t1 = 10.2%, *p* = 0.481) were the most frequent preventives, followed by onabotulinumtoxinA (t0 = 2.5%, t1 = 4.2%, *p* = 0.063), only in chronic migraine patients, flunarizine (t0 = 2.1%, t1 = 4.6%, *p* = 0.065), lamotrigine (t0 = 0.7%, t1 = 1.4%, *p* = 0.5), gabapentin (t0 = 0.7%, t1 = 1.1%, *p* = 1.0), valproic acid (t0 = 0.7%, t1 = 0.7%, p = 1.0). The percentage of anti-CGRP or anti-CGRP receptor monoclonal antibodies were galcanezumab (t0 = 8.5%, t1 = 12.8%, *p* = 0.017), erenumab (t0 = 6.0%, t1 = 7.4%, *p* = 0.125) and fremanezumab (t0 = 5.3%, t1 = 6.0%, *p* = 0.5). There was no serious adverse event reported for all NSAIDs, triptans, and prophylactic therapies.

### Determinants of NSAID consumption and efficacy

**Figure 1:**
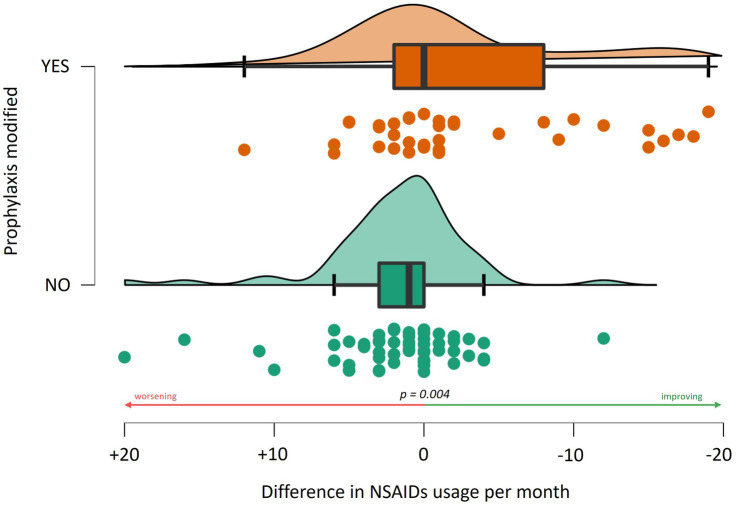
Association between the change in the monthly frequency of NSAID use and adjustments to prophylactic treatment regimens.

**Figure 2:**
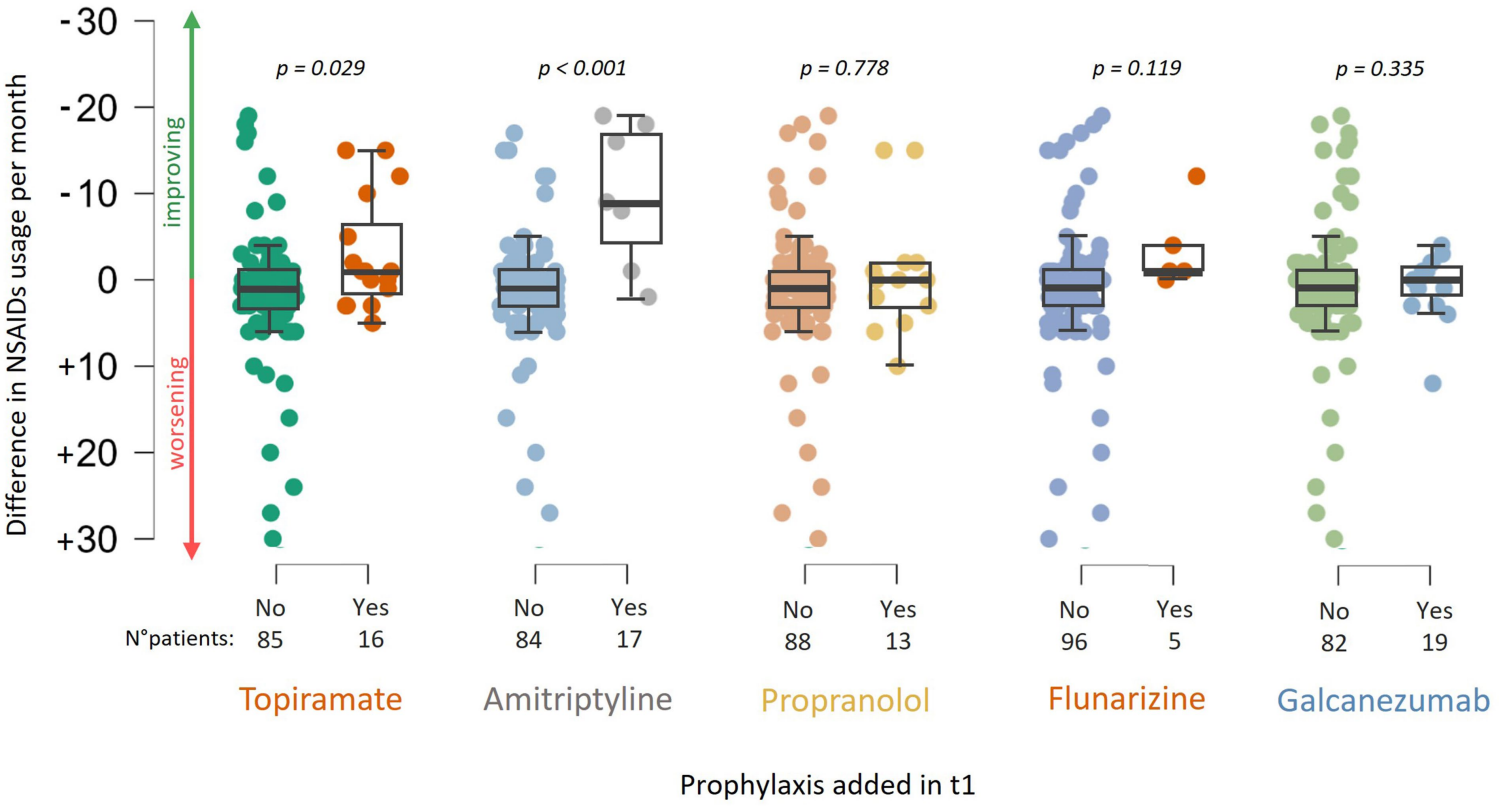
Association between the change in NSAID usage frequency and the addition of prophylactic medications at t1, including: topiramate, amitriptyline, propranolol, flunarizine, and galcanezumab.

There was no significant difference in NSAID use related to age, gender, or the presence of (visual) aura. At baseline (t0), patients who had never undergone a specialist consultation for headache (naïve patients) exhibited a higher frequency of NSAID usage compared to patients who had previously been examined for headache, i.e., by their general practitioner or at second level but not at third level headache centers (12.1 ± 8.9 vs. 10.0 ± 10.0, W = 1709.000, *p* = 0.051). Between t0 and t1, there was a reduction in NSAID intake frequency (10.6/month vs. 6.1/month, *p* < 0.001). A significant association was observed between a change or a start in prophylaxis and a reduction in NSAID usage frequency, particularly indicating that patients changing prophylaxis experienced a monthly reduction of 1.8 ± 9.2 administrations (63 patients). In comparison, those not changing had an increase of 3.1 (± 7.6) monthly administrations (*W* = 834.000, *p* = 0.004, 38 patients, Figure 1). Specifically, the addition of topiramate between t0 and t1 resulted in a reduction of 3.45 (± 6.2) administrations per month (*W* = 443.500, *p* = 0.029, addition of topiramate at t1 vs. no addition of topiramate at t1), while amitriptyline was associated with a reduction of 9.8 (±8.2) administrations per month (*W* = 90.500, *p* < 0.001). In a limited number of patients, monoclonal therapy was introduced between t0 and t1 before undergoing our centre examination, which precluded the ability to perform a statistical evaluation. This analysis was solely conducted for Galcanezumab, which did not demonstrate a correlation with a reduction in NSAID consumption (see Figure 2).

The efficacy of NSAID treatment at t0 was evaluated in 116 patients, with pain freedom in 55.2%, pain relief in 29.3%, and no effect of NSAIDs in 15.5% of cases. At t1, data from 63 patients showed pain complete resolution in 79.4%, partial resolution in 11.1%, and no resolution in 9.5% of cases. Inferential analysis demonstrated a statistically significant correlation between patients changing prophylaxis and those experiencing effective improvement in efficacy between t0 and t1 (*X*^2^ = 4.722, *p* = 0.03). Notably, amitriptyline (*X*^2^, *p* = 0.04) and propranolol (*X*^2^ = 8.797, *p* = 0.01) showed greater efficacy in improving the therapeutic response to NSAIDs.

### Determinants of headache frequency

**Figure 3:**
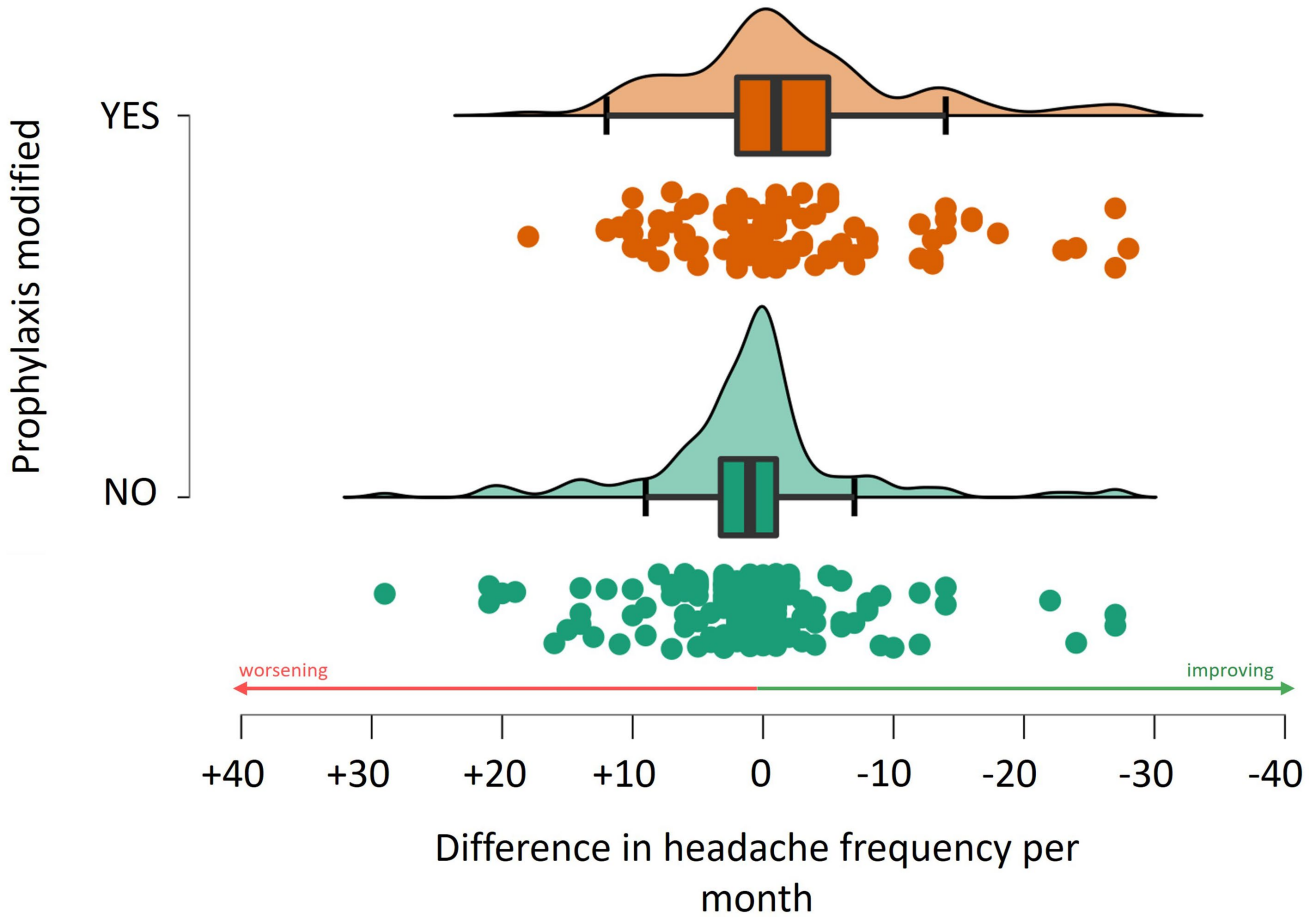
Association between the change in the monthly frequency of headache and adjustments to prophylactic treatment regimens.

Analysis revealed that a change in prophylaxis was associated with a reduction in headache frequency by approximately 2.1 (± 8.8) days (*W* = 5960.5, *p* = 0.003, see Figure 3). The most effective prophylaxis in reducing headaches seemed to be linked to the addition of topiramate (reduction of 2.9 ± 7.2 days, *W* = 2636.5, *p* = 0.006) and amitriptyline with a reduction of 1.6 ± 6.9 days (*W* = 3773.5, *p* = 0.025). No statistically significant differences were observed in those adding monoclonal antibodies anti-CGRP, considering the limited number who actually initiated monoclonals between t0 and t1.

### Impact of prophylactic changes on chronic and episodic migraine management

**Figure 4:**
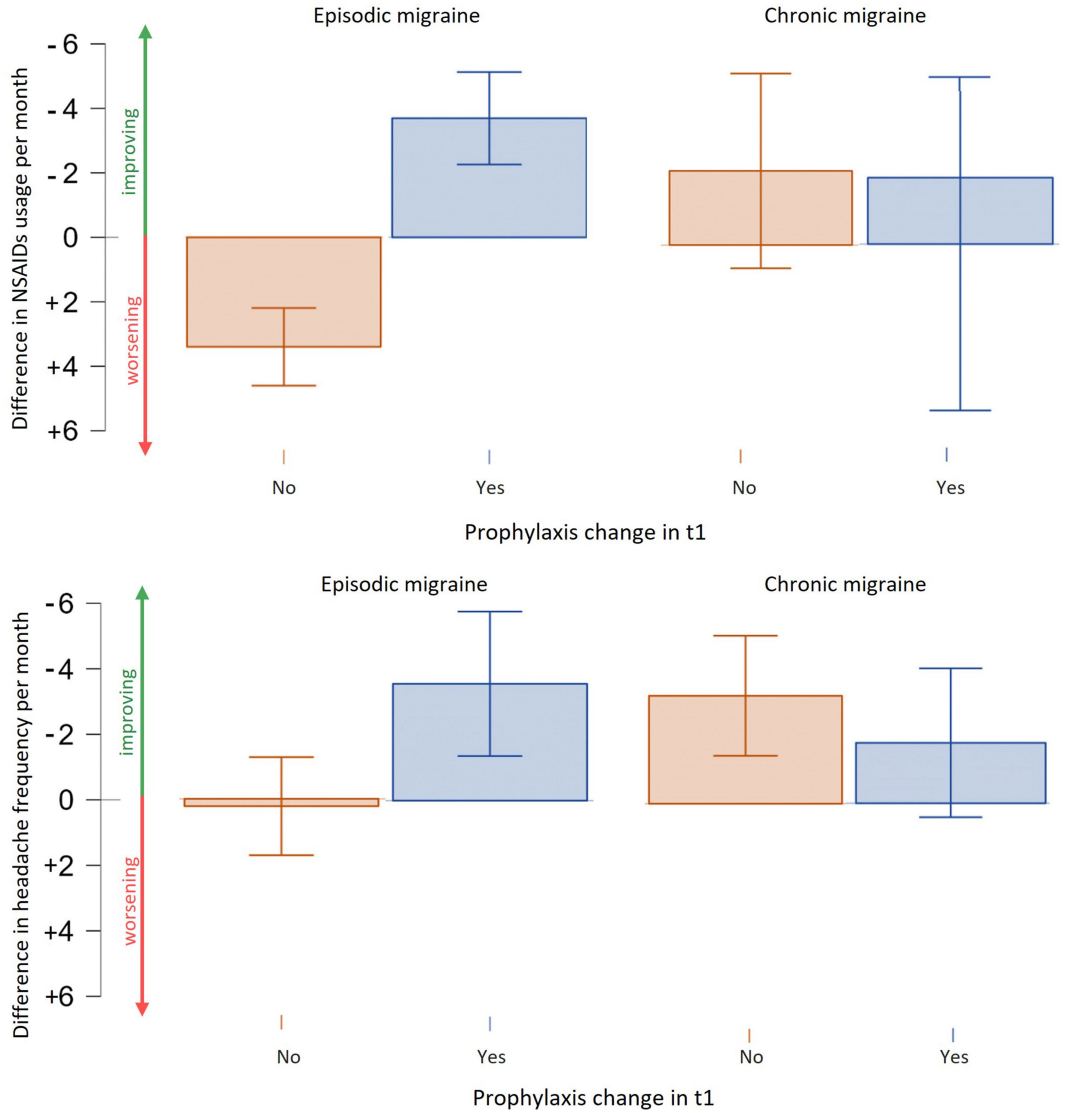
Efficacy of prophylactic therapies change in chronic and episodic migraine. Episodic migraine patients experience a reduction in terms of NSAIDs usage per month and days of headache per month with a change or addition of prophylaxis. Chronic migraine patients showed a trend in reduction of days of migraine and NSAIDs usage but not statistically significant.

There was no significant difference in age between chronic and episodic migraine (CM and EM, respectively) patients. Chronic patients exhibited higher headache frequency (19.1 ± 6.9 vs. 7.82 ± 5.5 *p* < 0.001) and NSAID consumption (18.1 ± 11.94 vs. 6.9 ± 5.67, *p* < 0.001) compared to episodic ones. Prophylactic therapies at t0 were administered in 38.2% of EM patients and in 64.5% of CM ones. The MIDAS scores were also significantly higher in chronic than in episodic patients (78.35 ± 58.09 vs. 32.42 ± 31.20, *p* < 0.001). Upon follow-up, a reduction in headache frequency (chronic: −9.52 ± 7.11, *p* < 0.001 vs. episodic: −5.89 ± 4.18, *p* < 0.001), NSAID intake (−8.3 ± 7.95, *p* < 0.001 vs. −5.20 ± 4.99, *p* < 0.001) and MIDAS (−31.21 ± 29.17 *p* < 0.001 vs. −22.15 ± 24.37, *p* < 0.001) was observed with no differences in the entity of reduction between chronic and episodic groups (*p* > 0.05). However, the change in prophylaxis therapy in t1 did not significantly reduce headache frequency (prophylaxis change: −1.88 ± 5.75 days/month vs. −3.31 ± 6.07, *p* = 0.267) or NSAID usage (−2.16 ± 11.93 NSAIDs/month vs. +2.44 ± 6.44, *p* = 0.702) in CM patients. In contrast, EM patients who underwent prophylactic changes showed a significant reduction in monthly NSAID consumption (−3.69 ± 7.31 NSAIDs/month vs. +3.40 ± 8.08, W = 295,000, *p* < 0.001) and days of headache per month (−3.56 ± 8.00 days/month vs. +0.22 ± 9.3, W = 3,073, *p* = 0.006) (see Figure 4).

## Discussion

The analysis conducted in the present study demonstrated the prevalent use of nonspecific acute medications for migraine, such as NSAIDs and simple analgesics, attending for the first-time tertiary headache center care. At baseline (t0), 58.7% of patients were using NSAIDs and simple analgesics, a figure that decreased to 46.6% at the follow-up (t1), 3 months after our first examination. Conversely, the percentage of patients taking triptans increased from 65.1% at t0 to 72.8% at t1, a trend that is corroborated by similar previous studies. For instance, the study by Cevoli et al. (19), which involved 953 patients from 10 Italian headache centers diagnosed with migraine, showed a predominant use of NSAIDs (66%), paracetamol (8.5%), and triptans (17%). Similarly, Brusa et al. (11) observed a higher prescription rate of nonspecific medications such as NSAIDs (52.4%) compared to triptans (33.7%). Baratta, in a study of 4,424 primary headache cases, noted the use of NSAIDs in 53.7% of cases and triptans in 12.9%, with a greater propensity for NSAIDs’ use among patients without a migraine diagnosis (16). Affaitati et al. (20) examined therapeutic changes post-consultation, noting a shift from a predominant use of NSAIDs (80%) to triptans in 53% of cases, with a return to NSAIDs after 1 year in 56% of patients who had switched treatments.

Differently from the above studies, we found a high utilization of triptans (65.1% patients at t0 and 72.8% at t1), likely due to the third-level nature of the center and the continuity of care for patients already undergoing treatment prescribed at primary and secondary care levels. Furthermore, there is a notable prevalence of prophylactic therapy prescription, from 42.5% at t0 to 65.2% at t1 (half of patients with chronic migraine), compared to the national average, in contrast to Cevoli’s findings of only 1.5% of patients on prophylactic therapy (19). The outcomes of the inferential study underscore the significance of introducing or modifying prophylactic therapy in reducing headache frequency, decreasing the use of NSAIDs and simple analgesics, and enhancing their efficacy. This also translates into a fourfold reduction in rate of medication overuse (MO), dropping from 13.7 to 3.8%.

The sub-analyses revealed that episodic migraine patients experienced a notable reduction in both the number of headache days and NSAIDs per month with the change or addition of prophylactic therapy. In contrast, chronic patients exhibit a more complex response to prophylactic therapy changes. In fact, while there was a slight reduction in NSAID intake and in headache frequency in some cases, the overall benefit was not statistically significant. This limited response might be attributed to the inherent challenges in managing chronic migraine, which are often compounded by NSAID overuse (21). Higher disability and NSAID usage per month, even when the prophylactic therapies were more frequent at t0, can make the change in preventives ineffectiveness in this cohort of patients. These data underscored the complexity of treatment in this subgroup and the importance of tailored therapeutic strategies at the highest level of care to optimize outcomes for CM sufferers.

Following worldwide accepted guidelines, it is appropriate to use NSAIDs (including aspirin) and non-opioid analgesics, i.e., paracetamol, for mild-to-moderate attacks and migraine-specific agents (i.e., primarily triptans) for moderate or severe attacks and mild-to-moderate attacks poorly responders to NSAIDs (22). The commitment of a specialized headache center is to prescribe patients the specific treatment for the headache appropriately and advise them to take medications proportionally to the pain intensity. Acute treatments have to be used virtually by everyone with migraine when an attack occurs, with the aspirational goals of relieving pain and associated symptoms up to pain freedom proportionally to the intensity of symptoms while restoring function with minimal side effects.

However, even if headache is the most common neurological syndrome, recent epidemiological studies have shown that more than half of patients with migraine have never consulted a doctor or have received a correct diagnosis (23). Furthermore, in Italy, many migraineurs make a wrong self-diagnosis (24). This explains why the majority of people with migraine make use of self-prescribed medication, taking over-the-counter analgesics to treat their headache, often without consulting a doctor (19, 25). The lack of proper treatment for headache, however, can lead to an overuse of acute pain-relief medicines and medication overuse headache (MOH), which makes chronic migraine even more disabling (18).

In this light, patients’ education is essential: clinicians must explain the migraine condition to patients and the principles of its effective and safe management, including advice on the correct use of acute medications, potential adverse effects, and the importance of preventing medication overuse (9). People with migraine who need to take acute treatment on a regular basis should be instructed to limit medication use to an average of two headache days per week, and those who exceed this limit should be offered a preventive treatment (22). In addition to patient education, a greater understanding of the pathophysiology of migraine and consequently the knowledge about its most effective and safe treatments by general practitioners would greatly benefit patients management even before they enter a tertiary center.

There are still diverse unmet needs in the acute migraine management. Some patients are non-responders to triptans and/or NSAIDs or present contraindications such as high vascular risk in pregnancy, older ages, and so on. In this light, having at disposal new drugs, effective and well-tolerated at the same time, could help increase the proportion of patients who may have a good response to acute migraine treatments.

The findings from the present study emphasize the value of a specialized approach in headache management. They demonstrate that therapeutic adjustments with increased use of specific medications and prophylactic therapies may result in a reduced intake of analgesics, which correlates with a decrease in the average frequency of headaches and, consequently, an improvement in patients’ disability and quality of life.

### Limitations of the study

The retrospective design of this study underlies its present limitations. The first limitation is the absence of medications such as gepants and lasmiditan, which have been recently approved for the symptomatic treatment of migraine and were not present in our sample. Consequently, it was not possible to assess the impact of these drugs on headache frequency and NSAID usage. Another limitation arises from the statistical analyses performed on unbalanced patient groups, which may reduce the accuracy of the results. This limitation should be viewed in the context of a real-world study that aims to describe as realistically as possible the characteristics of patients at a tertiary care center.

## Data Availability

The raw data supporting the conclusions of this article will be made available by the authors, without undue reservation.
